# Supplementary material for: East Gobi megalake systems reveal East Asian Monsoon dynamics over the last interglacial-glacial cycle
Source: Nat Commun. 2023 Apr 13;14:2103. doi: 10.1038/s41467-023-37859-1 (PMC10102015; doi:10.1038/s41467-023-37859-1)
Supplement: Supplementary file 1 — Supplementary information [file 41467_2023_37859_MOESM1_ESM.pdf]

**Supplementary information for**

# East Gobi megalake systems reveal East Asian Monsoon dynamics over the last interglacial-glacial cycle

Hongwei Li[1], Xiaoping Yang[1*], Louis Anthony Scuderi[2], Fangen Hu[3], Peng Liang[1], Qida Jiang[4], Jan-Pieter Buylaert[5], Xulong Wang[6], Jinhua Du[7], Shugang Kang[6], Zhibang Ma[4], Lisheng Wang[4], Xuefeng Wang[4]

[1] Key Laboratory of Geoscience Big Data and Deep Resource of Zhejiang Province, School of Earth Sciences, Zhejiang University, Hangzhou 310058, China.

[2] Department of Earth and Planetary Sciences, University of New Mexico, Albuquerque, NM 87131, USA.

[3] Geographical Research Center, Yichun University, Yichun 336000, China.

[4] Key Laboratory of Cenozoic Geology and Environment, Institute of Geology and Geophysics, Chinese Academy of Sciences, Beijing 100029, China.

[5] Department of Physics, Technical University of Denmark, DTU-Risø campus, Frederiksborgvej 399, 4000 Roskilde, Denmark.

[6] State Key Laboratory of Loess and Quaternary Geology, Institute of Earth Environment, Chinese Academy of Sciences, Xi'an 710061, China.

[7] School of Earth Science and Resources, Chang'an University, Xi'an 710054, China

* Corresponding author. Email: xpyang@zju.edu.cn

**Contents of this file**

Supplementary Figures 1-14

Supplementary Tables 1-5

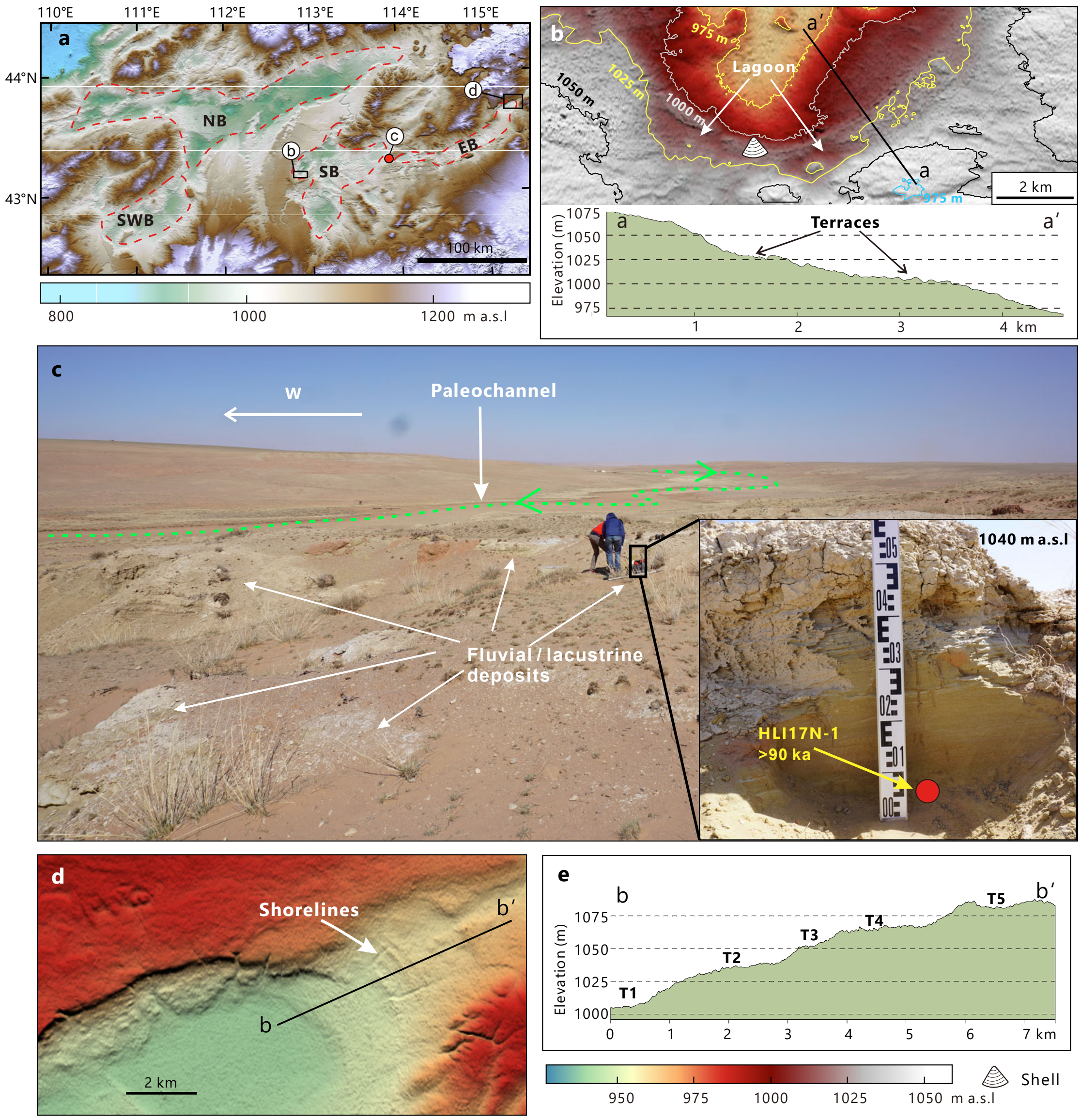


**Supplementary Fig. 1. Geomorphic evidence of the paleolakes in the SB and EB of the East Gobi Desert. (a)** Topography of the study area derived from the SRTM 90 m resolution DEM elevation data. Black boxes and red circles delineate areas of panels b-d. **(b)** The AW3D (30 m resolution) DEM shows a shoreline barrier system along the southwest edge of the SB (upper panel). In the lower panel the cross profile (a-a') shows terraces at elevations of 1025 m and 1008 m**. (c)** Fluvial/ lacustrine deposit outcrops and the OSL sampling site (inset photo) along the southern bank of the paleochannel connecting the EB and SB. The quoted quartz OSL age is >90 ka because the $D_e$ value is 185±28 Gy (≥ 150 Gy). The elevation of lacustrine deposit remnants ranges from ~1030-1045 m, corresponding with T2 in panel **e**. It indicates the EB might have a lake level ca. 20 m higher than the SB during MIS 5 prior to the outflow river incising a 30

m deep valley. **(d-e)** Shoreline terraces and a cross profile (b-b') at the northeast side of the EB shown using AW3D DEM data.

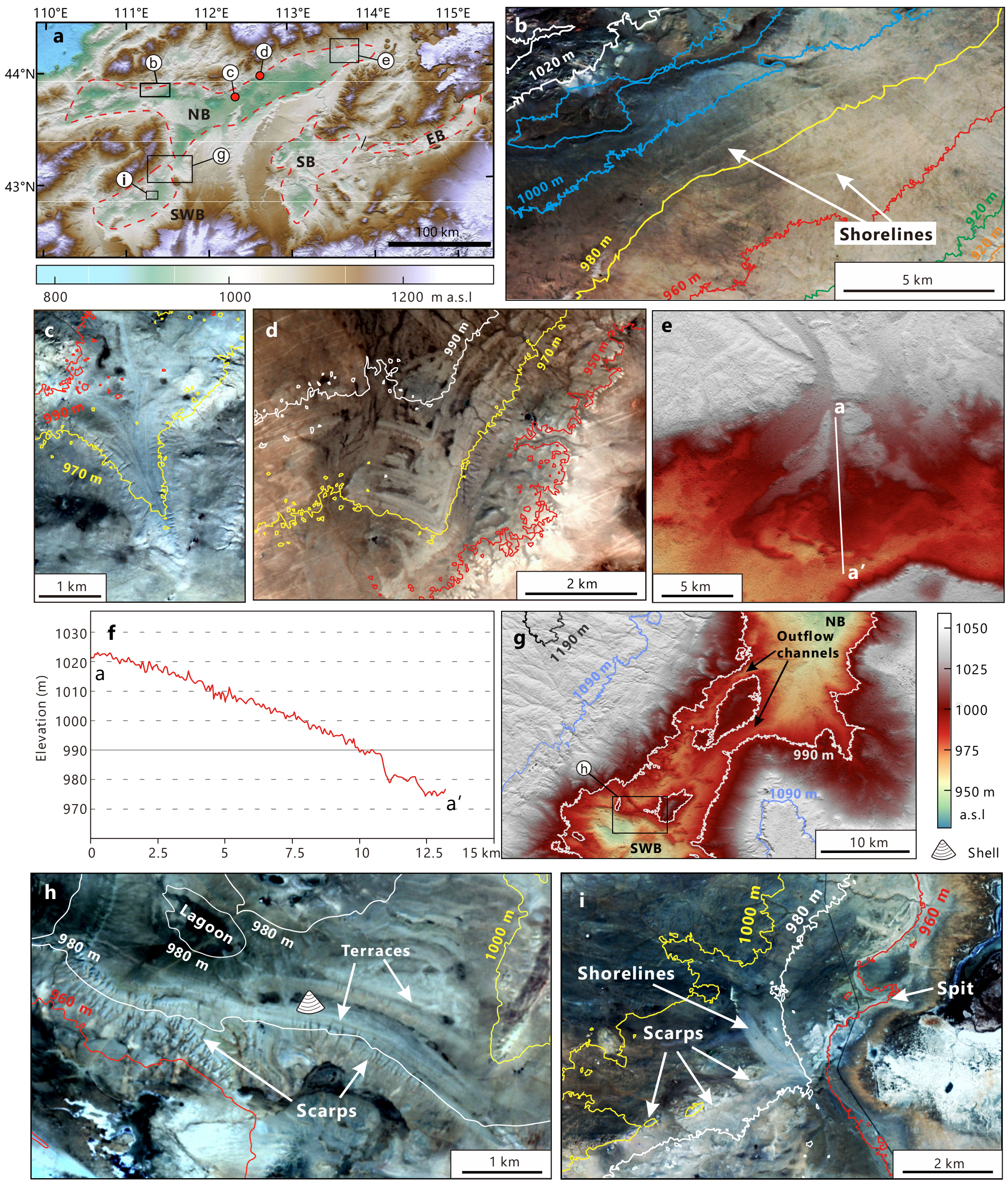


**Supplementary Fig. 2. Geomorphic evidence of the paleolakes in the NB and SWB of the East Gobi Desert. (a)** Topography of the study area derived from the SRTM DEM. Black boxes and red circles delineate areas of panels b-e and g-i. **(b-d)** Sentinel-2A satellite images showing beach shorelines, cuspate spit and cuspate foreland of NB. **(e-f)** Fan-delta cross profile (a-a') at the northeast part of NB. Note the profile has a slope break at ~990 m. **(g)** AW3D DEM data showing the topography of the area between the SWB and the NB. **(h-i)** The shoreline barrier system and beach features at the north and western margin of the SWB.

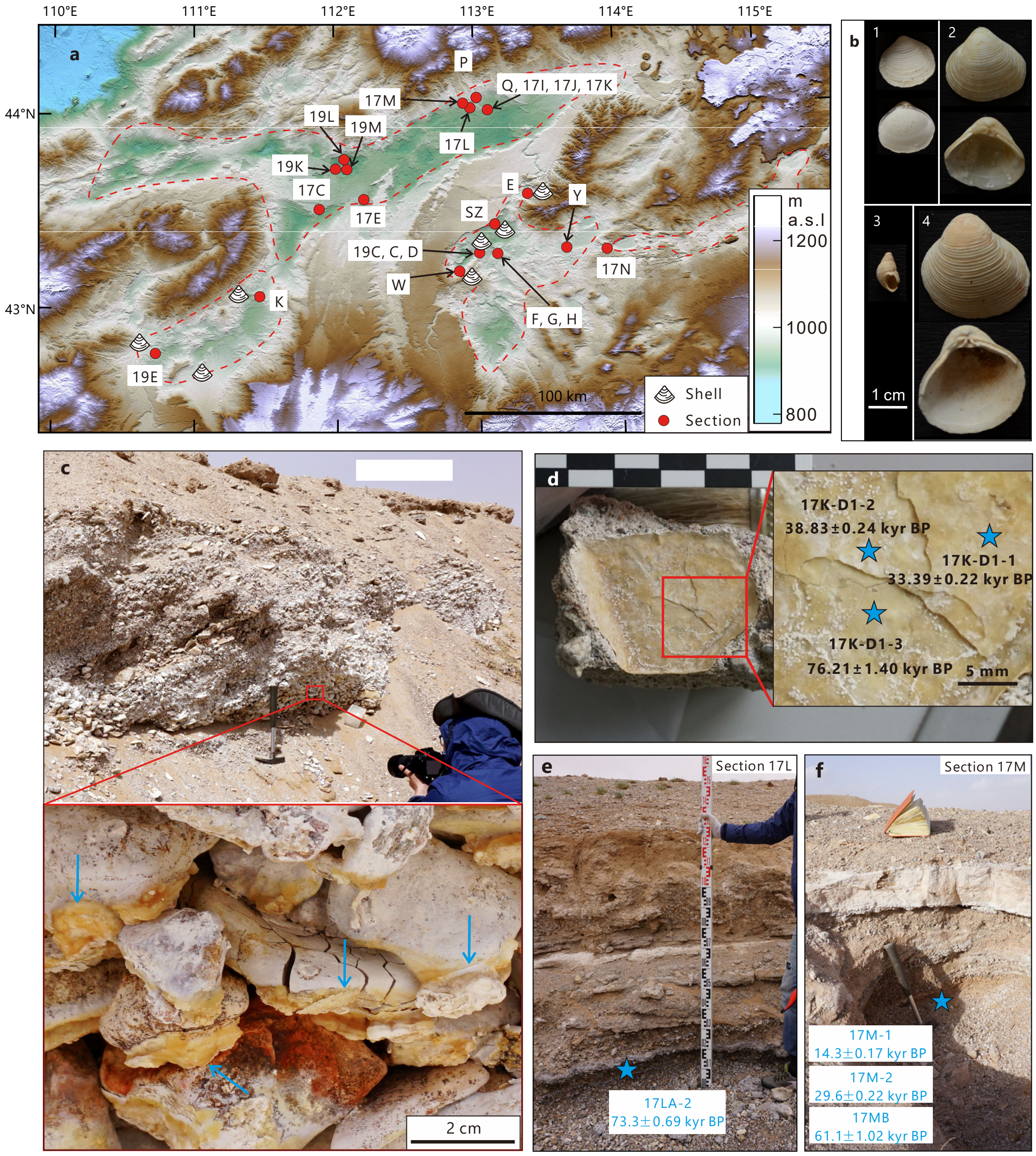


**Supplementary Fig. 3. Fossil evidence of the paleolake system and carbonate coatings on the shoreline deposits**. **(a)** Topography of the study area. Sections for analysis in this study and mollusk fossil sites are also shown. **(b)** Mollusk shell species found in the study area. 1. *Corbicula Lenuis* (Clessin, 1887); 2. *Corbicula Largillierti* (Philippi, 1844); 3. *Parafossarulus striatulus* (Benson, 1842); 4. *Corbicula Fluminea* (Müller, 1774). **(c)** Photo showing shoreline deposits and carbonate coatings precipitated at the bottom of the gravels (taken from section 17C, see panel a for position). **(d)** Photo showing the parallel lamination (~0.5 mm in thickness) and U series ages of the carbonate coatings collected from site 17K (spit deposits, see panel a for location). Note the coating layers are also weakly cemented with each other, while

tightly adhering to the sands beneath it. **(e-f)** Stratigraphy and chronology (U series ages of carbonate coatings) of sections 17L and 17M, both of which are from the same spit as section 17K shown in panel d.

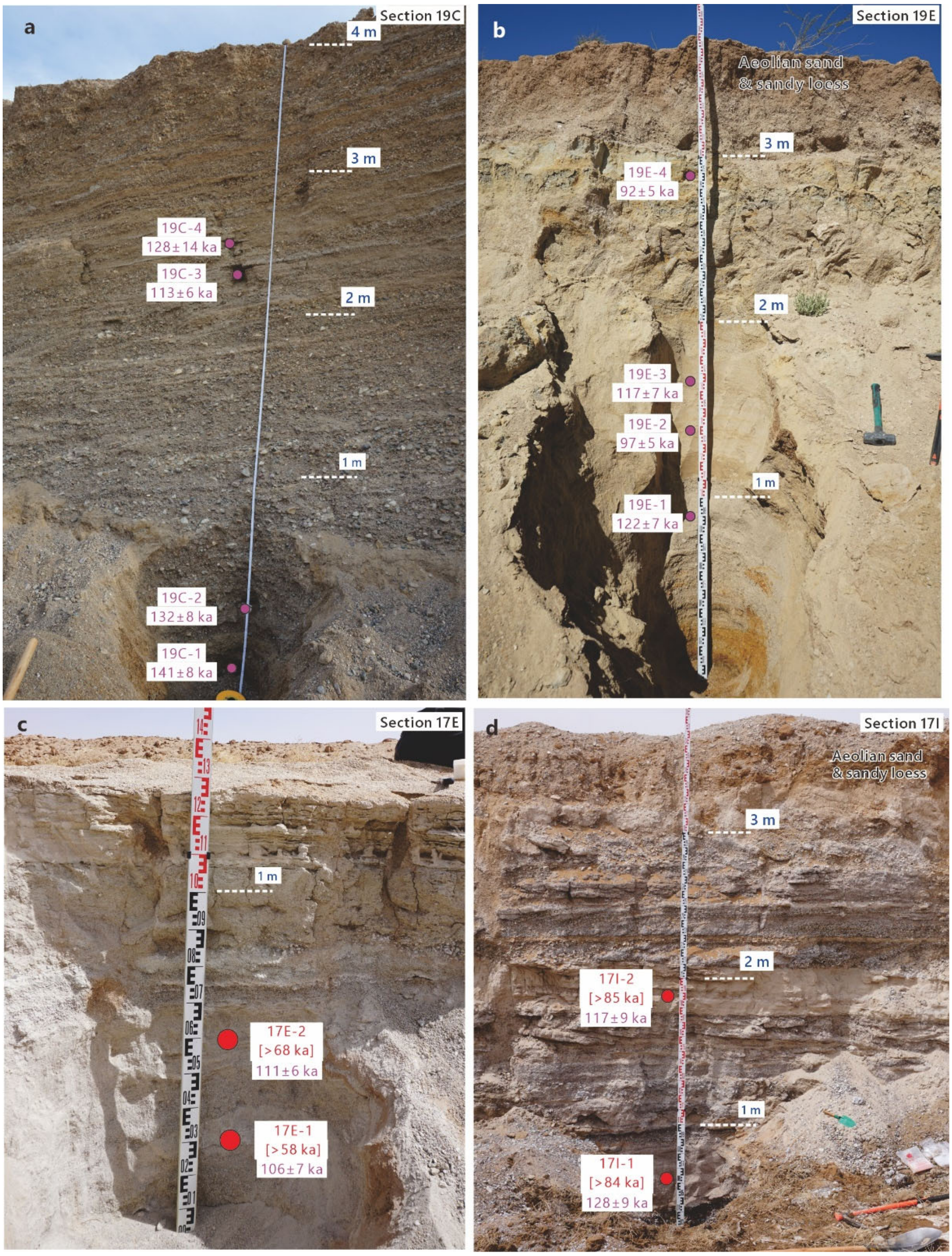


**Supplementary Fig. 4. Stratigraphy and luminescence ages of shoreline deposits (see Supplementary Fig. 3a for positions).** Quartz OSL ages with $D_e$ value ≥150 Gy are enclosed in square brackets and indicate minimum ages, while the K-feldspar pIRIR ages are shown in purple. **(a)** Shoreline deposits and chronology of section 19C, which is ~60 m away from Section C (Fig. 3b). **(b)** Fan-delta deposits in the SWB (Fig. 2f) and chronology. **(c-d)** Quaretz OSL and K-feldspar pIRIR luminescence chronology of spit deposits at Section 17E and 17I.

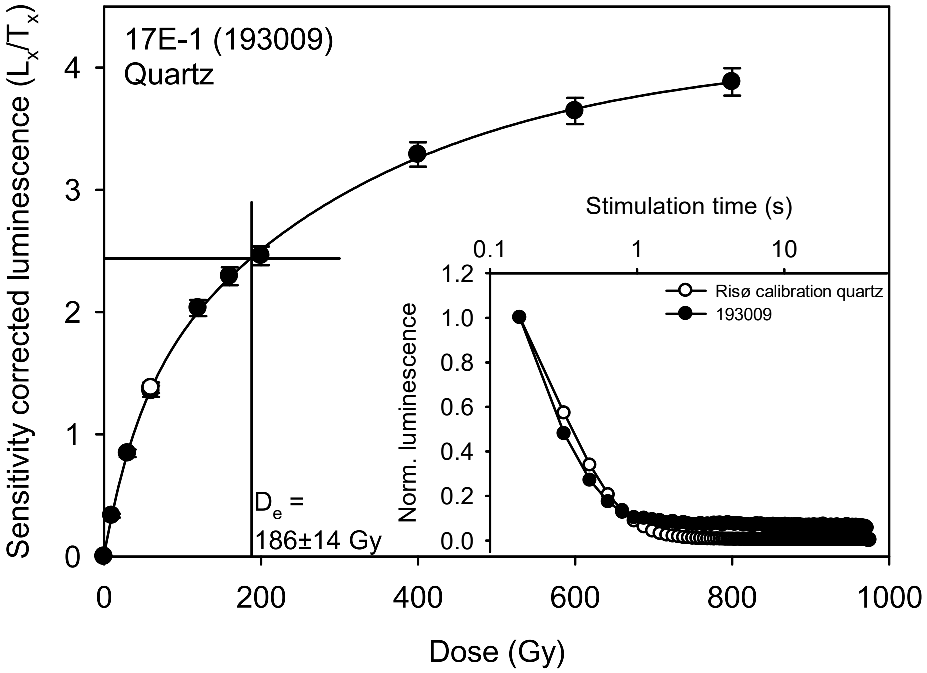


**Supplementary Fig. 5. Quartz OSL dose response curve for an aliquot (8 mm) of quartz from sample 17E-1.** The response curve was fitted with a sum of two saturating exponential functions. Recycled point shown as open symbol. The natural luminescence signal is not in saturation on the laboratory dose response curve but it is well-known that quartz OSL equivalent dose measurements often underestimate when $D_e$'s >150 Gy are measured. The inset shows a regenerative dose decay curve signal together with that of Risø calibration quartz which is known to be dominated by a fast component (data normalized to first datapoint). The quartz OSL signal from the samples in this study is dominated by the fast component but has a contribution from medium-slower components; the contribution of the latter components is reduced by using early background subtraction.

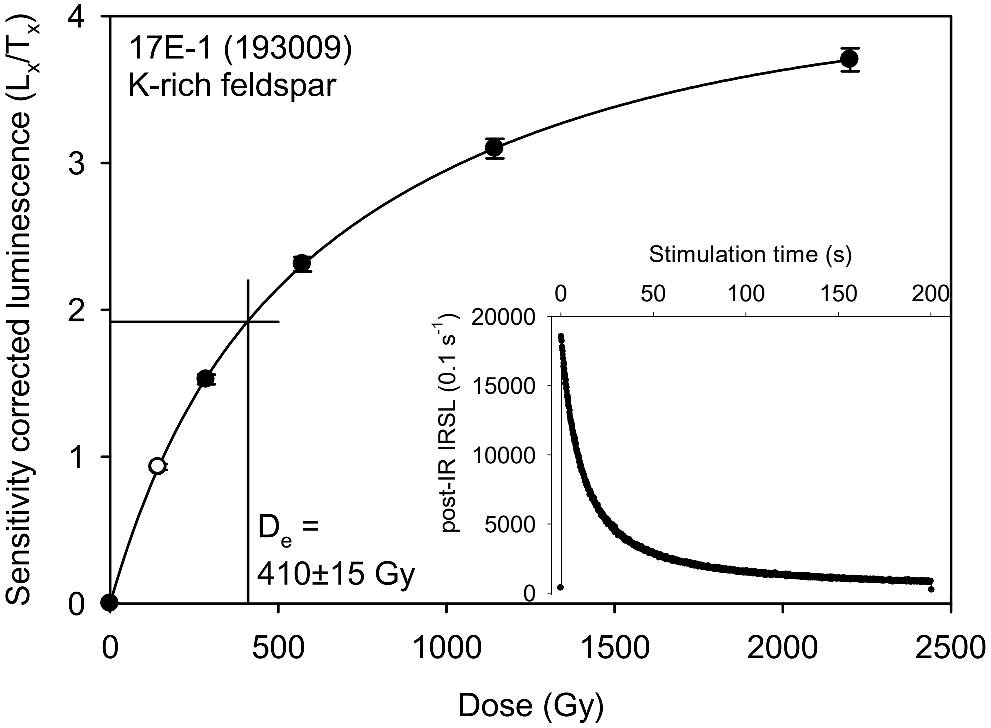


**Supplementary Fig. 6. Post-IR IRSL ($pIRIR_{200,290}$) dose response curve for an aliquot (2 mm) of K-rich feldspar from sample 17E-1.** Curve was fitted with a sum of two saturating exponential functions. Recycled point is shown as an open symbol and it can be seen that the natural luminescence signal lies well below the laboratory saturation level. The test dose size is 135 Gy and the inset shows the $pIRIR_{200,290}$ signal from the test dose measured in the first (natural) measurement cycle.

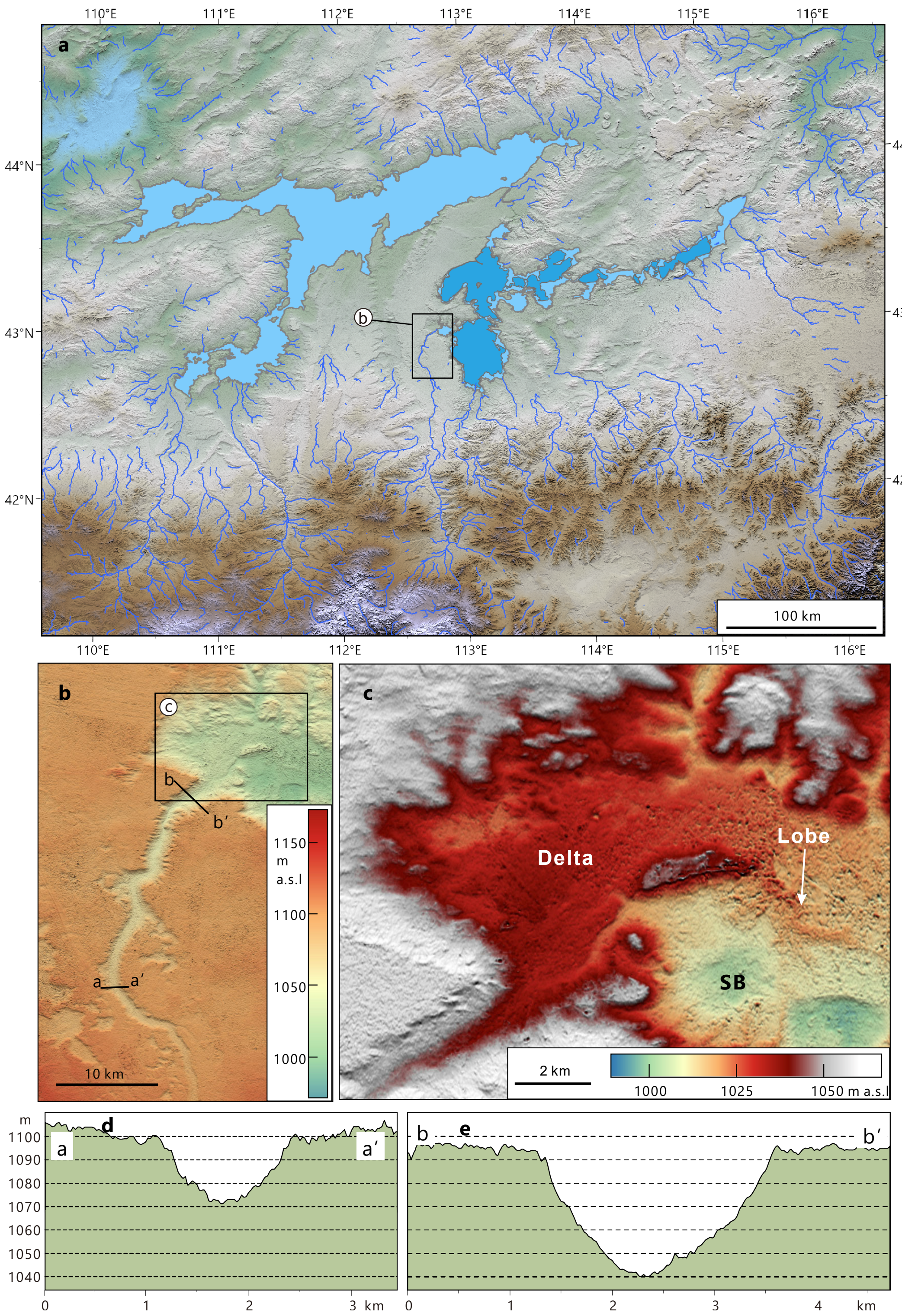


**Supplementary Fig. 7. Fluvial network in the East Gobi Desert. (a)** Paleodrainage (blue lines) identified in the ALOS AW3D DEM data. Reconstructed paleolakes during MIS 5 and the Holocene are also shown in light and dark blues, respectively. **(b)** DEM data shows lower reach of a paleochannel that flowed into the SB. **(c)** Details of the river mouth shown by DEM data. Note the delta lobe has an elevation ~ 1025 m,

corresponding exactly with the highest lake level of the SB. **(d-e)** Cross profiles show paleochannel size, indicating previously it was a large river.

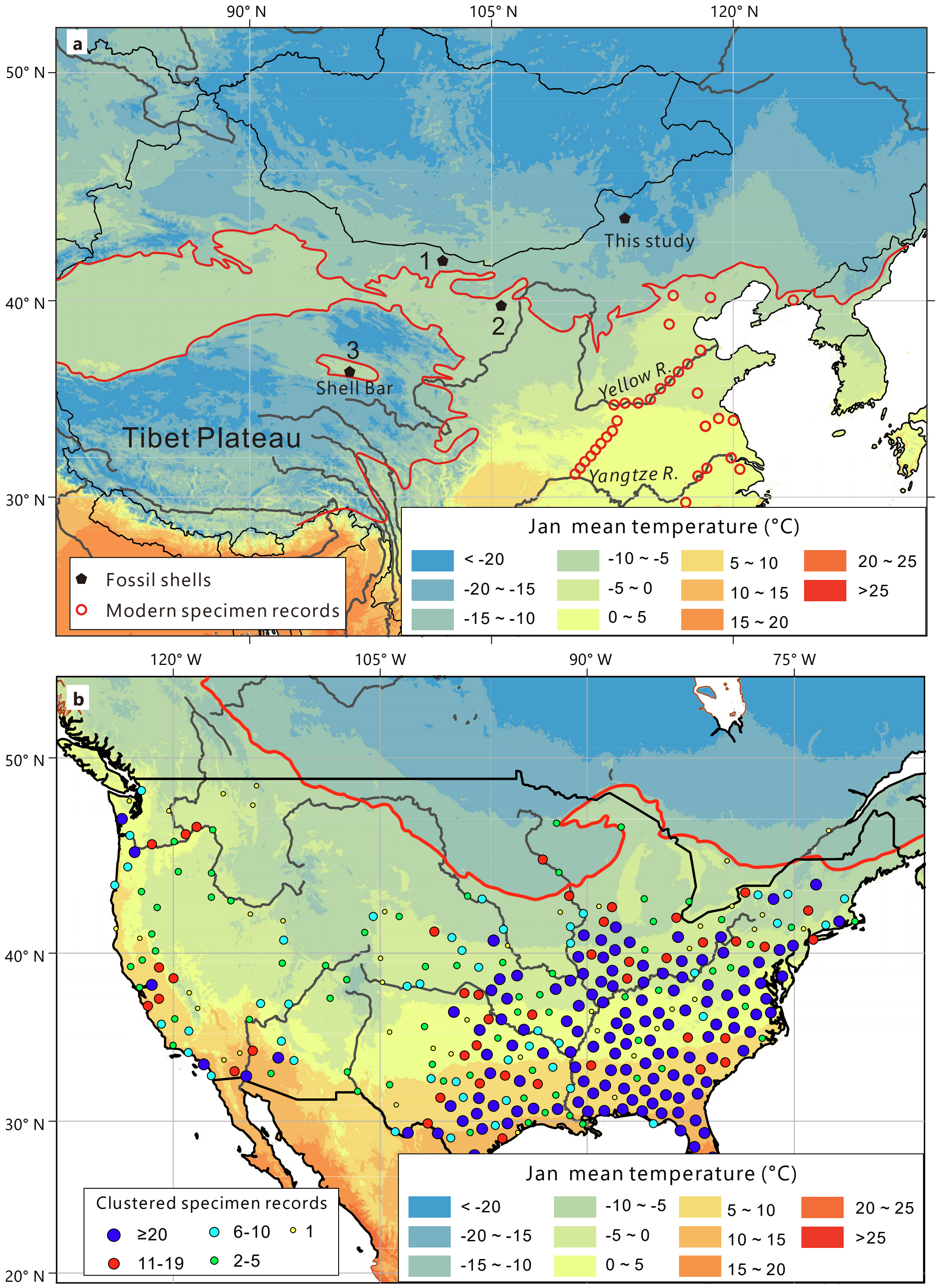


**Supplementary Fig. 8. Records of Corbicula fluminea in North China (a) and North America (b) projected on a map of January (coldest month) temperature.** The January mean temperature for 1970-2000 is obtained from WorldClim Version 2.0, available at http://www.worldclim.com/version2. The -10 °C isotherm is highlighted with a red line on the maps. Black pentagons in (a) represent the sites with *Corbicula fluminea* shells dated to the late Pleistocene. Site 1 is Sogu Nur. The initial radiocarbon

dating of the shell from the beach ridge indicates that it belongs to MIS 3[1], while the recent luminescence dating suggests the beach formed during MIS 5[2]. Site 2 is Mega lake Jilantai-hetao[3], which is believed to have filled before 50-60 ka based on OSL ages; MET-pIRIR (multiple-elevated-temperature post-infrared infrared-stimulated luminescence) dates the mega lake to MIS 5[4]. Site 3 is a shell bar in Qaidam Basin. The radiocarbon ages of the shells fall within MIS 3[5]. Again, the luminescence dating suggested the shell bar formed during MIS 5[6]. Records of modern *Corbicula fluminea* in mid and north China obtained from the references[7-12], are shown by red circles. The *Corbicula fluminea* data of North America was obtained from the website of the United States Geological Survey (https://nas.er.usgs.gov/viewer/omap.aspx?SpeciesID=92).

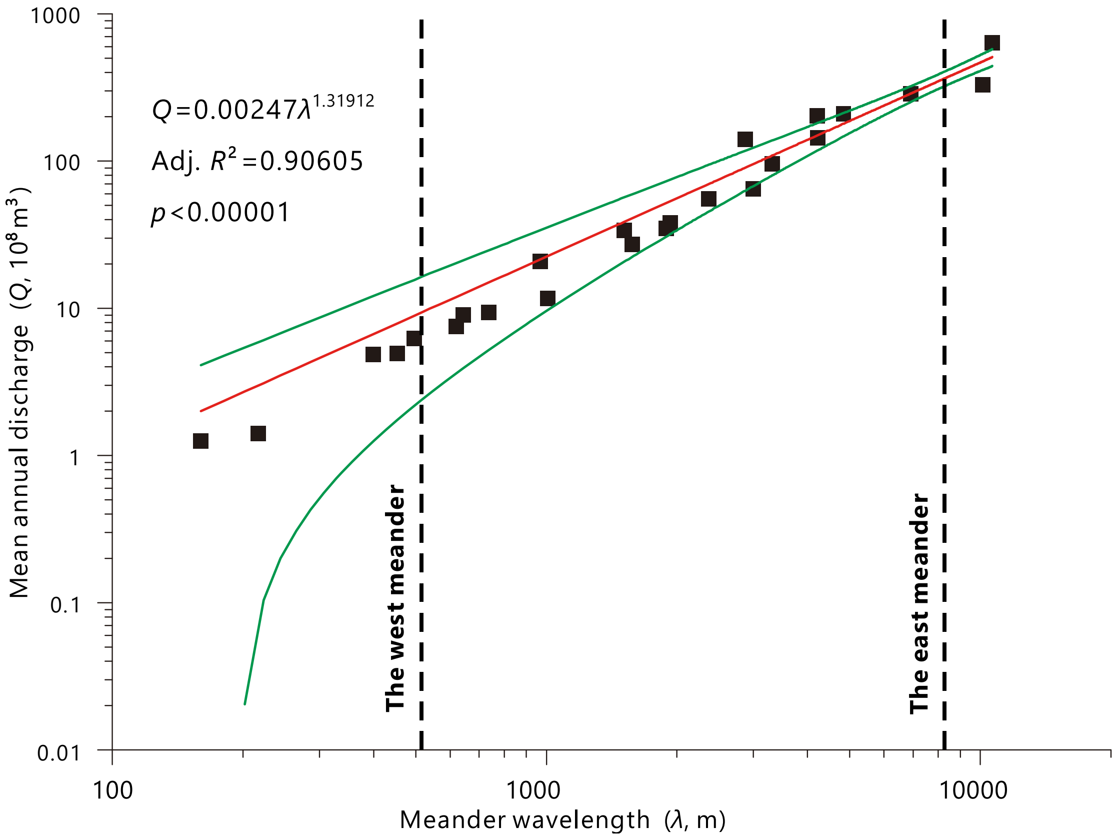


**Supplementary Fig. 9. Scatter plot of modern meander wavelength against mean annual discharge in China.** Red line represents a power trendline, while the 95% confidence interval is shown by the green lines (equation shown in the plot, see Supplementary Table 5 for data). Meander channels in the study area are also shown by the dashed lines.

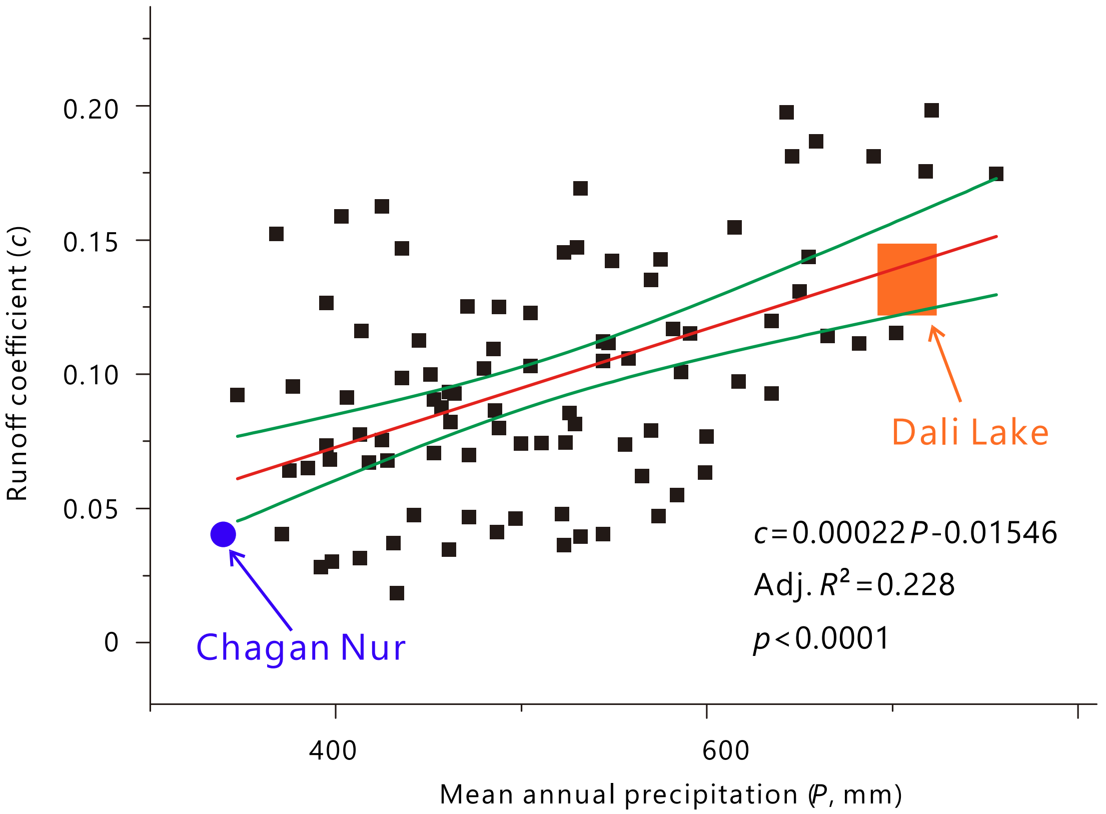


**Supplementary Fig. 10. Regression between runoff coefficient (c) and mean annual precipitation (P) for 92 modern catchments of North China, with 95% confidence interval shown by the green lines.** The precipitation and runoff coefficients of Chagan Nur[13] and Dali Lake[14] during Holocene high-stand periods, both of which are located in the East Gobi Desert, are also plotted on this chart (blue circle and orange square, respectively). The regression line with 95% confidence level contains the two lakes data, indicating the empirical equation works well in the East Gobi Desert. Data source: Yang et al. (2007)[15].

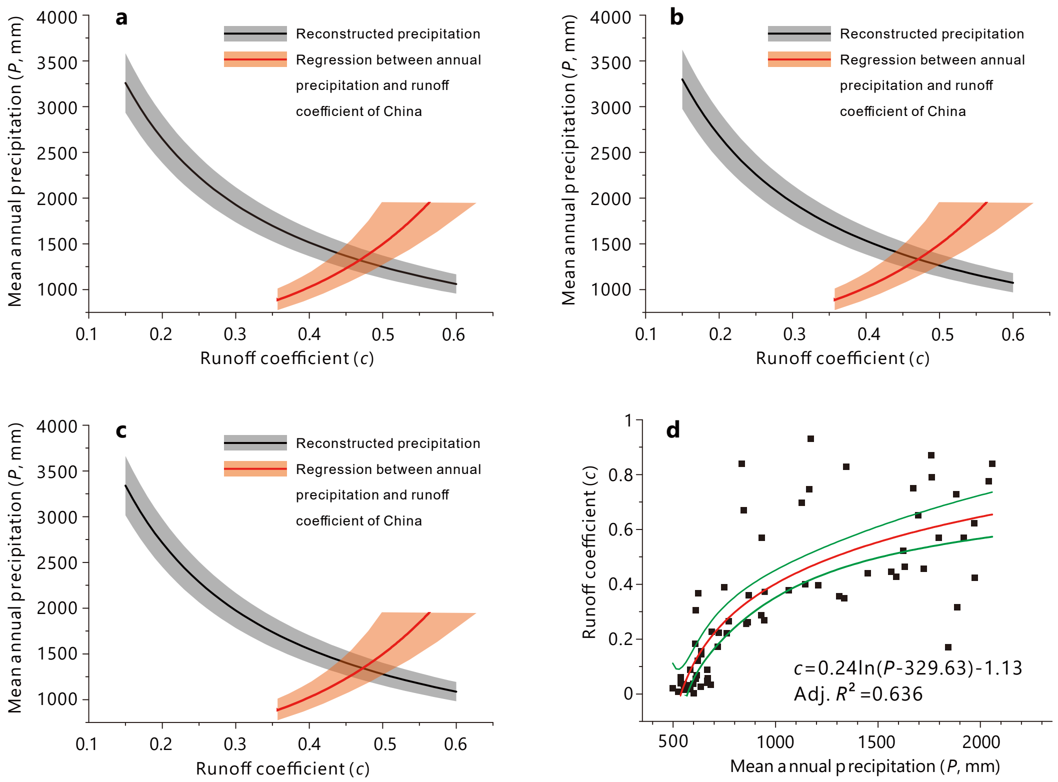


**Supplementary Fig. 11. (a-c)** Annual precipitation reconstruction of an earlier humid period, during which the meander between NB and SB formed, estimated by a water balance model with different lake evaporation rates: **(a)** 900 mm/year, **(b)** 1000 mm/year, **(c)** 1100 mm/year. The black line represents the reconstructed precipitation at different runoff coefficients, with 95% confidence interval shown by the shaded area. The regression between mean annual precipitation and runoff coefficient from 73 catchments of China (red line, 95% confidence interval shown by the shaded area) provides a possible constraint on the precipitation of the humid period. **(d)** Regression of mean annual precipitation and runoff coefficient from 73 catchments of China (with 95% confidence interval shown by the green lines) displays a logarithmic relationship. Data source: Huo et al. (2021)[16].

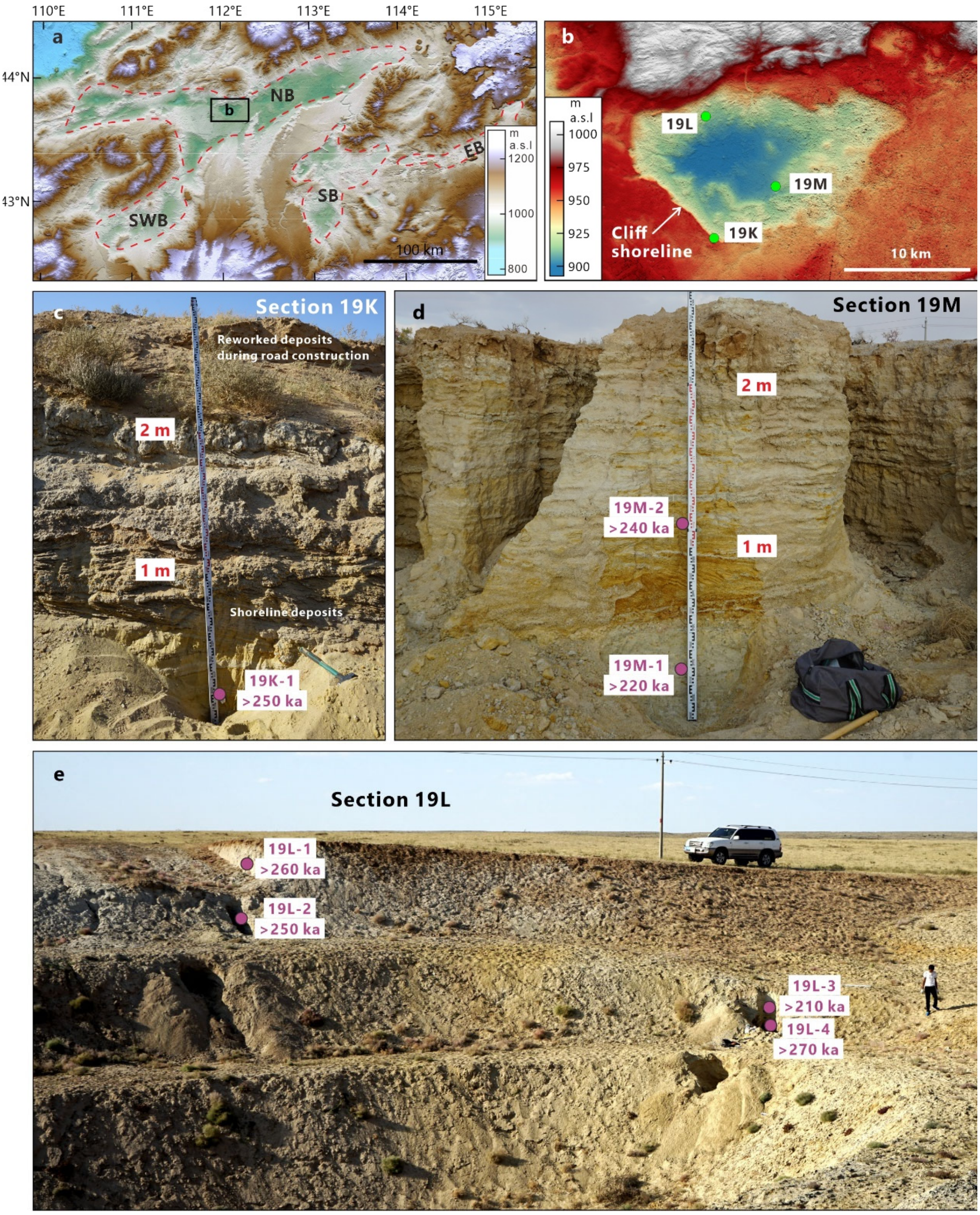


**Supplementary Fig. 12. Luminescence chronology (minimum ages) of the lake deposits from the NB. (a)** Topography of the study area. **(b)** AW3D DEM data showing the sections' positions and surrounding topography. Note that section 19K is located at the shoreline cliff. **(c)** Lake-floor deposits and chronology of section 19M. **(d)** Section 19M of the lake-floor cliff and its chronology. **(e)** Section 19L and its chronology.

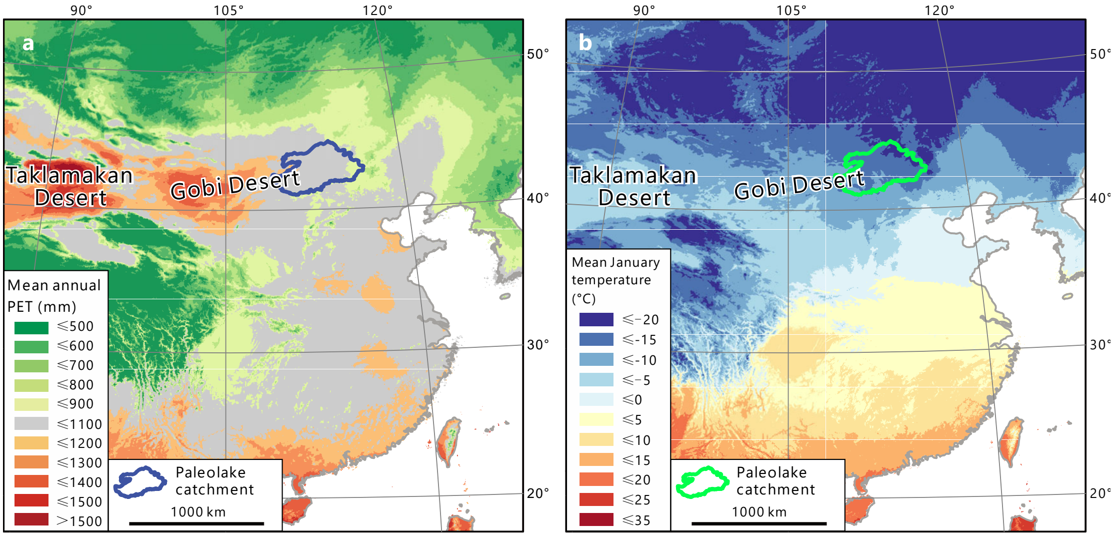


**Supplementary Fig. 13. Mean annual potential evapotranspiration (PET, a) and mean January temperature (b) over the East Gobi Desert and surrounding area.** Note that the area with mean annual PET between 900-1100 mm is marked in gray. Data source: The temperature data (1970-2000) is from WorldClim Version 2.0, available at http://www.worldclim.com/version2; the PET data (1981-2010) is from TerraClimate[17].

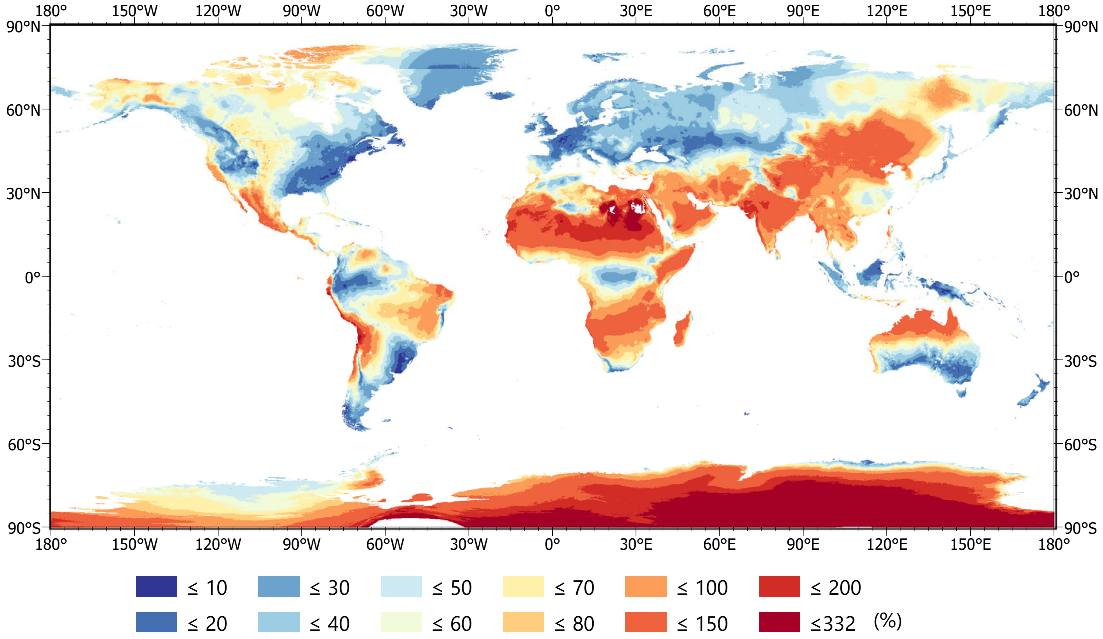


**Supplementary Fig. 14. World map showing the coefficient of variation of monthly precipitation** (Monthly precipitation data from WorldClim Version 2.0, available at http://www.worldclim.com/version2).

**Supplementary Table 1. Shell radiocarbon ages**

| Sample ID | Section name | Lab ID | $\delta^{13}C$ (PDB) | Conventional radiocarbon age | Calibrated age (±2σ) | pMC (%) |
|---|---|---|---|---|---|---|
| C3a | C | Beta-427880 | −6.5‰ | 42430±660 BP | 46860–44620 BP | 0.5±0.1 |
| D1a | D | Beta-427881 | −5.4‰ | >43500 BP | NA | <0.4 |
| E3 | E | Beta-427882 | −5.0‰ | >43500 BP | NA | <0.4 |
| G1 | G* | Beta-427883 | −4.7‰ | 38040±370 BP | 42705–41975 BP | 0.9±0.1 |

*The shell from section G was not collected from the fresh section but from the ground 5 m away from the section. We speculate the shell may have been eroded from the section, reworked and then transported to the site.

## Supplementary Table 2. Uranium series ages of shells and carbonate coatings

| Sample ID | Section name | Dating material | $^{238}U$ (ppb) | $^{232}Th$ (ppt) | $^{230}Th$ / $^{232}Th$ (atomic x$10^{-6}$) | $\delta^{234}U$ * (measured) | $^{230}Th$ / $^{238}U$ (activity) | $^{230}Th$ age (yr) (uncorrected) | $^{230}Th$ age (yr) (corrected) | $\delta\ ^{234}U_{Initial}$ † (corrected) | $^{230}Th$ age (yr BP) ‡ (corrected) |
|---|---|---|---|---|---|---|---|---|---|---|---|
| C2 | C | Shell | 3941 ±10 | 49166 ±992 | 1343.7 ±27.3 | 460.8 ±2.3 | 1.0168 ±0.0038 | 118932 ±834 | 118711 ±847 | 644 ±4 | 118645 ±847 |
| C3b | C | Shell | 3570 ±11 | 57569 ±1162 | 815.4 ±16.6 | 388.4 ±2.4 | 0.7975 ±0.0032 | 88586 ±584 | 88272 ±623 | 498 ±3 | 88206 ±623 |
| D1b | D | Shell | 2092 ±4 | 13538 ±272 | 2722.8 ±55.0 | 390.9 ±2.1 | 1.0684 ±0.0030 | 143250 ±902 | 143131 ±905 | 585 ±3 | 143065 ±905 |
| E1 | E | Shell | 2586 ±5 | 6496 ±131 | 7945.9 ±160.6 | 488.2 ±2.1 | 1.2108 ±0.0035 | 157734 ±1062 | 157692 ±1062 | 762 ±4 | 157626 ±1062 |
| E4 | E | Shell | 4297 ±12 | 467 ±10 | 171769.3 ±3636.7 | 528.7 ±2.4 | 1.1318 ±0.0041 | 131285 ±945 | 131283 ±945 | 766 ±4 | 131217 ±945 |
| ET | E | Carbonate coating | 10238 ±43 | 123083 ±2516 | 801.1 ±16.7 | 423.2 ±2.0 | 0.5842 ±0.0035 | 55952 ±440 | 55718 ±469 | 495 ±2 | 55651 ±469 |
| G1 | G | Shell | 8092 ±31 | 151908 ±3082 | 797.6 ±16.4 | 259.6 ±2.4 | 0.9081 ±0.0047 | 130247 ±1324 | 129848 ±1348 | 374 ±4 | 129782 ±1348 |
| K | K | Shell | 2509 ±4 | 5805 ±117 | 7020.4 ±141.5 | 530.5 ±2.2 | 0.9852 ±0.0021 | 104016 ±431 | 103977 ±432 | 711 ±3 | 103912 ±432 |
| SZ | SZ | Shell | 2932 ±4 | 20767 ±416 | 2603.9 ±52.4 | 454.9 ±2.0 | 1.1187 ±0.0023 | 142078 ±690 | 141955 ±695 | 679 ±3 | 141890 ±695 |
| W | W | Shell | 3570 ±5 | 20228 ±405 | 2985.3 ±60.0 | 484.3 ±2.0 | 1.0259 ±0.0020 | 117218 ±479 | 117119 ±484 | 674 ±3 | 117054 ±484 |
| Q | Q | Carbonate coating | 5647 ±20 | 4557 ±92 | 8454.2 ±173.5 | 446.9 ±2.6 | 0.4137 ±0.0020 | 36068 ±217 | 36052 ±217 | 495 ±3 | 35987 ±217 |
| 17JC-1 | 17J | Carbonate coating | 41665 ±243 | 31150 ±644 | 3745.9 ±80.0 | 429.2 ±3.2 | 0.1699 ±0.0013 | 13712 ±120 | 13697 ±120 | 446 ±3 | 13630 ±120 |
| 17JC-2 | 17J | Carbonate coating | 19336 ±86 | 254888 ±5209 | 638.2 ±13.3 | 427.8 ±2.4 | 0.5102 ±0.0031 | 47071 ±369 | 46813 ±410 | 488 ±3 | 46746 ±410 |
| 17KA-1 | 17K | Carbonate coating | 18921 ±87 | 85573 ±1756 | 796.7 ±16.8 | 389.8 ±2.7 | 0.2185 ±0.0014 | 18508 ±136 | 18415 ±151 | 411 ±3 | 18348 ±151 |
| 17KA-2 | 17K | Carbonate coating | 18916 ±85 | 11593 ±237 | 10026.1 ±209.6 | 408.4 ±2.6 | 0.3727 ±0.0023 | 33006 ±248 | 32994 ±248 | 448 ±3 | 32927 ±248 |
| 17KA | 17K | Carbonate coating | 12945 ±178 | 26110 ±634 | 2404.1 ±67.1 | 394.2 ±1.8 | 0.2941 ±0.0057 | 25546 ±555 | 25505 ±555 | 424 ±2 | 25438 ±555 |
| 17KD-1 | 17K | Carbonate coating | 12764 ±49 | 28613 ±582 | 2784.5 ±57.6 | 412.2 ±2.3 | 0.3786 ±0.0020 | 33503 ±219 | 33458 ±221 | 453 ±3 | 33391 ±221 |
| 17KD-2 | 17K | Carbonate coating | 11687 ±42 | 15237 ±309 | 5446.4 ±112.0 | 412.4 ±2.2 | 0.4307 ±0.0021 | 38927 ±240 | 38901 ±240 | 460 ±2 | 38834 ±240 |

| Sample ID | Section name | Dating material | $^{238}U$ (ppb) | $^{232}Th$ (ppt) | $^{230}Th$ / $^{232}Th$ (atomic x$10^{-6}$) | $\delta^{234}U$ * (measured) | $^{230}Th$ / $^{238}U$ (activity) | $^{230}Th$ age (yr) (uncorrected) | $^{230}Th$ age (yr) (corrected) | $\delta\ ^{234}U_{Initial}$ † (corrected) | $^{230}Th$ age (yr BP) ‡ (corrected) |
|---|---|---|---|---|---|---|---|---|---|---|---|
| 17KD2-1 | 17K | Carbonate coating | 24880 ±258 | 35535 ±800 | 4235.9 ±105.0 | 381.5 ±2.0 | 0.3669 ±0.0054 | 33171 ±563 | 33141 ±563 | 419 ±2 | 33074 ±563 |
| 17KD-3 | 17K | Carbonate coating | 7838 ±70 | 199511 ±4367 | 478.0 ±11.3 | 417.1 ±2.3 | 0.7378 ±0.0093 | 76768 ±1363 | 76279 ±1398 | 517 ±4 | 76212 ±1398 |
| 17LA-2 | 17L | Carbonate coating | 21577 ±101 | 21457 ±440 | 12216.0 ±256.7 | 459.5 ±2.6 | 0.7368 ±0.0048 | 73357 ±686 | 73338 ±686 | 565 ±3 | 73271 ±686 |
| 17M-1 | 17M | Carbonate coating | 15533 ±117 | 25918 ±553 | 1667.3 ±37.8 | 360.8 ±2.2 | 0.1687 ±0.0018 | 14354 ±164 | 14319 ±166 | 376 ±2 | 14252 ±166 |
| 17M-2 | 17M | Carbonate coating | 19249 ±85 | 64082 ±1311 | 1624.3 ±34.0 | 360.4 ±2.3 | 0.3280 ±0.0020 | 29710 ±217 | 29640 ±222 | 392 ±2 | 29573 ±222 |
| 17MB | 17M | Carbonate coating | 12693 ±113 | 87787 ±1922 | 1498.4 ±35.4 | 426.8 ±1.8 | 0.6285 ±0.0079 | 61270 ±1013 | 61137 ±1016 | 507 ±3 | 61070 ±1016 |
| 17C-A2-1 | 17C | Carbonate coating | 23920 ±202 | 48041 ±1041 | 3565.7 ±82.8 | 388.8 ±2.7 | 0.4343 ±0.0052 | 40147 ±575 | 40106 ±575 | 435 ±3 | 40039 ±575 |
| 17C-A2-2 | 17C | Carbonate coating | 45586 ±366 | 102400 ±2197 | 3853.4 ±87.9 | 387.4 ±2.9 | 0.5250 ±0.0059 | 50598 ±718 | 50553 ±718 | 447 ±3 | 50486 ±718 |
| 17C-A2-3 | 17C | Carbonate coating | 38197 ±303 | 12759 ±274 | 22396.2 ±510.0 | 381.3 ±3.1 | 0.4537 ±0.0050 | 42582 ±577 | 42575 ±577 | 430 ±4 | 42508 ±577 |
| 17C-A1-1 | 17C | Carbonate coating | 21999 ±171 | 32979 ±706 | 5373.7 ±122.3 | 399.6 ±2.4 | 0.4886 ±0.0053 | 45802 ±618 | 45772 ±618 | 455 ±3 | 45705 ±618 |
| 17C-B2-1 | 17C | Carbonate coating | 14917 ±62 | 29780 ±606 | 4987.2 ±103.4 | 419.8 ±2.6 | 0.6038 ±0.0034 | 58564 ±451 | 58525 ±451 | 495 ±3 | 58458 ±451 |
| 17CC1-1B | 17C | Carbonate coating | 12450 ±70 | 6142 ±128 | 21961.9 ±472.1 | 396.7 ±2.4 | 0.6571 ±0.0052 | 66936 ±721 | 66926 ±721 | 479 ±3 | 66859 ±721 |
| 17CC1-1 | 17C | Carbonate coating | 13735 ±130 | 71505 ±1583 | 1961.7 ±47.3 | 388.3 ±1.9 | 0.6194 ±0.0083 | 62428 ±1106 | 62325 ±1107 | 463 ±3 | 62258 ±1107 |
| 17CC1-2 | 17C | Carbonate coating | 29760 ±184 | 23892 ±498 | 9376.1 ±203.4 | 388.2 ±2.7 | 0.4565 ±0.0039 | 42630 ±451 | 42614 ±451 | 438 ±3 | 42547 ±451 |
| 17CC1-3 | 17C | Carbonate coating | 32553 ±170 | 4862 ±100 | 55161.7 ±1169.2 | 391.8 ±2.9 | 0.4997 ±0.0036 | 47412 ±437 | 47409 ±437 | 448 ±3 | 47342 ±437 |
| 17CC2-1 | 17C | Carbonate coating | 24238 ±254 | 24562 ±555 | 9632.4 ±239.9 | 389.6 ±1.9 | 0.5920 ±0.0088 | 58789 ±1130 | 58769 ±1129 | 460 ±3 | 58702 ±1129 |
| 17CD1-1 | 17C | Carbonate coating | 24060 ±146 | 19721 ±411 | 10055.6 ±217.9 | 387.3 ±2.6 | 0.4999 ±0.0042 | 47632 ±508 | 47616 ±508 | 443 ±3 | 47549 ±508 |

*$\delta^{234}U = ([^{234}U/^{238}U]_{activity} - 1) \times 1000$.

† $\delta^{234}U$ initial was calculated based on $^{230}Th$ age (T), i.e., $\delta^{234}U_{initial} = \delta^{234}U_{measured} \times e^{\lambda 234 \times T}$. Corrected $^{230}Th$ ages assume an initial $^{230}Th/^{232}Th$ atomic ratio of $4.4 \pm 2.2 \times 10^{-6}$. These are values for a material at secular equilibrium, with the bulk earth $^{232}Th/^{238}U$ value of 3.8. Errors are arbitrarily assumed to be 50%.

‡ B.P. stands for "Before Present" where the "Present" is defined as the year 1950 A.D.

**Supplementary Table 3. Quartz OSL dating results of the lacustrine and aeolian deposits from the Xi'an laboratory (China). Uncertainties are all given at 1 σ.**

| Sample ID | Section name | Depth (m) | Quartz age (ka) | Water content (%) | U (ppm) | Th (ppm) | K (%) | Quartz dose rate (Gy/ka) | Quartz $D_e$ (Gy) | Number of aliquots (n) |
|---|---|---|---|---|---|---|---|---|---|---|
| **C-1** | C | 6.25 | 87 ± 4 | 15 ± 5 | 1.76±0.01 | 2.28±0.08 | 1.27±0.03 | 1.67 ± 0.06 | 145 ± 5 | 10 |
| **C-2** | C | 4.9 | 81 ± 4 | 10 ± 5 | 1.04±0.02 | 2.73±0.01 | 1.13±0.00 | 1.53 ± 0.06 | 123 ± 3 | 10 |
| **D-1** | D | 5.15 | >66 * | 10 ± 5 | 0.93±0.06 | 3.91±0.06 | 2.43±0.04 | 2.73 ± 0.11 | 180 ± 3 | 10 |
| **D-2** | D | 4.7 | >67 * | 10 ± 5 | 2.05±0.03 | 10.33±0.04 | 1.96±0.03 | 2.95 ± 0.11 | 198 ± 7 | 10 |
| **D-3** | D | 3.75 | >62 * | 10 ± 5 | 1.13±0.01 | 6.80±0.06 | 2.32±0.03 | 2.87 ± 0.11 | 178.2 ± 1.3 | 10 |
| **D-4** | D | 2.8 | >74 * | 10 ± 5 | 1.09±0.01 | 4.48±0.05 | 2.5±0.02 | 2.90 ± 0.12 | 213 ± 2 | 10 |
| **D-5** | D | 0.35 | 5.1 ± 0.2 | 8 ± 4 | 1.96±0.05 | 9.13±0.21 | 1.92±0.06 | 3.00 ± 0.09 | 15.2 ± 0.4 | 10 |
| **F-1** | F | 0.7 | 10.7 ± 0.6 | 8 ± 4 | 0.82±0.02 | 3.52±0.08 | 1.91±0.02 | 2.36 ± 0.08 | 25.2 ± 1.0 | 10 |
| **G-1** | G | 3.45 | 12.9 ± 0.7 | 10 ± 5 | 0.92±0.02 | 5.08±0.06 | 2.02±0.02 | 2.46 ± 0.10 | 31.7 ± 1.2 | 10 |
| **G-2** | G | 2.9 | 6.7 ± 0.5 | 10 ± 5 | 0.75±0.01 | 2.72±0.06 | 2.37±0.00 | 2.60 ± 0.11 | 17.5 ± 1.1 | 10 |
| **G-3** | G | 2.5 | 6.7 ± 0.5 | 10 ± 5 | 0.98±0.05 | 3.12±0.09 | 2.24±0.02 | 2.57 ± 0.10 | 17.2 ± 1.0 | 10 |
| **G-4** | G | 1.9 | 6.5 ± 0.5 | 8 ± 4 | 0.89±0.04 | 3.16±0.11 | 2.04±0.04 | 2.44 ± 0.08 | 15.9 ± 1.1 | 10 |
| **G-5** | G | 1.3 | 6.2 ± 0.3 | 8 ± 4 | 0.97±0.02 | 2.71±0.03 | 2.21±0.02 | 2.58 ± 0.08 | 16.0 ± 0.5 | 10 |
| **H-1** | H | 1.95 | 11.2 ± 0.7 | 10 ± 5 | 1.37±0.01 | 7.79±0.13 | 2.13±0.02 | 2.85 ± 0.11 | 31.9 ± 1.5 | 10 |
| **H-2** | H | 1.5 | 6.8 ± 0.4 | 10 ± 5 | 0.89±0.03 | 3.65±0.03 | 2.05±0.01 | 2.43 ± 0.10 | 16.6 ± 0.6 | 10 |
| **H-3** | H | 0.8 | 6.6 ± 0.3 | 8 ± 4 | 1.66±0.09 | 5.09±0.13 | 2.07±0.02 | 2.78 ± 0.09 | 18.3 ± 0.7 | 10 |
| **Y-1** | Y | 4.2 | 9.2 ± 1.6 | 10 ± 5 | 0.71±0.01 | 2.15±0.04 | 2.57±0.05 | 2.71 ± 0.12 | 24.8 ± 4.2 | 19 |
| **Y-2** | Y | 2.1 | 8.9 ± 1.6 | 10 ± 5 | 0.91±0.02 | 2.55±0.11 | 2.43±0.02 | 2.69 ± 0.11 | 24.0 ± 4.2 | 20 |
| **17N-1** | 17N | 0.6 | >90 * | 10 ± 5 | 0.66±0.02 | 1.36±0.04 | 1.81±0.06 | 2.07 ± 0.09 | 185 ± 28 | 19 |
| **17I-1** | 17I | 3.3 | >86 * | 15 ± 5 | 1.78±0.01 | 2.33±0.04 | 1.88±0.03 | 2.24 ± 0.09 | 193 ± 22 | 18 |
| **17I-2** | 17I | 2 | >67 * | 8 ± 4 | 1.86±0.04 | 3.76±0.03 | 2.82±0.05 | 3.39 ± 0.12 | 228 ± 28 | 20 |

Notes:

(1) Ages denoted with an asterisk (*) are minimum ages because the $D_e$ values are ≥150 Gy. and K-feldspar $pIRIR_{200,290}$ ages (see Supplementary Table 4) are used for interpretation in the main text.

(2) Note that samples C1, C2, D1-D4, 17I-1 and 17I-2 (shaded gray) were also dated at the NLLD (Supplementary Table 4).

**Supplementary Table 4. Luminescence dating results of the lacustrine deposits from the Nordic Laboratory for Luminescence Dating (DTU, Denmark). Uncertainties are all given at 1 σ.**

| Sample ID | Section name | Risø lab code | Depth (m) | Quartz age (ka) | K-feldspar age (ka) | Water cont. (%) | $^{238}$U (Bq/kg) | $^{226}$Ra (Bq/kg) | $^{210}$Pb (Bq/kg) | $^{232}$Th (Bq/kg) | $^{40}$K (Bq/kg) | Quartz dose rate (Gy/ka) | K-feldspar dose rate (Gy/ka) | Quartz $D_e$ (Gy) | $n_r$ (Qz) | $n_a$ (Qz) | K-feldspar $D_e$ (Gy) | $n_r$ (Kf) | $n_a$ (Kf) |
|---|---|---|---|---|---|---|---|---|---|---|---|---|---|---|---|---|---|---|---|
| **P-1** | P | 193002 | 0.93 | >70 * | **93±5** | 7±5 | 21±7 | 29.8±1.5 | 22±8 | 30.3±1.1 | 933±25 | 3.55±0.15 | 4.49±0.16 | 272±12 | 0 | 12 | 424±9 | 0 | 9 |
| **Q-1** | Q | 193003 | 1.05 | >87 * | **104±6** | 13±5 | 50±8 | 30.0±1.8 | 39±9 | 15.2±1.2 | 850±27 | 2.78±0.11 | 3.72±0.13 | 278±16 | 1 | 14 | 428±10 | 0 | 8 |
| **17E-1** | 17E | 193009 | 0.95 | >58 * | **106±7** | 12±5 | 12±8 | 9.3±1.7 | 0±10 | 6.4±1.2 | 854±28 | 2.46±0.10 | 3.39±0.12 | 160±14 | 1 | 16 | 387±16 | 0 | 10 |
| **17E-2** | 17E | 193010 | 0.65 | >68 * | **111±6** | 12±5 | 6±5 | 6.8±1.0 | 7±6 | 7.3±0.7 | 970±19 | 2.72±0.10 | 3.66±0.12 | 208±17 | 1 | 10 | 441±12 | 0 | 10 |
| **19C-1** | 19C | 223011 | 1.60 | N.A. | **141±8** | 12±5 | 18±2 | 17.0±0.4 | 19±3 | 20.3±0.3 | 409±6 | 1.85±0.09 | 2.79±0.11 | N.A. | N.A. | N.A. | 393±16 | 2 | 11 |
| **19C-2** | 19C | 223012 | 1.85 | N.A. | **132±8** | 12±5 | 15±3 | 17.8±0.5 | 23±4 | 20.6±0.4 | 423±8 | 1.91±0.09 | 2.84±0.11 | N.A. | N.A. | N.A. | 376±16 | 1 | 9 |
| **19C-3** | 19C | 223013 | 3.80 | N.A. | **113±6** | 11±5 | 21±4 | 19.6±0.7 | 23±5 | 24.0±0.5 | 448±10 | 2.02±0.10 | 2.96±0.12 | N.A. | N.A. | N.A. | 335±11 | 0 | 9 |
| **19C-4** | 19C | 223014 | 4.10 | N.A. | **128±14** | 12±5 | 22±13 | 17.7±0.9 | N.A. | 21.8±1.1 | 395±15 | 1.80±0.09 | 2.73±0.11 | N.A. | N.A. | N.A. | 349±34 | 1 | 12 |
| **19E-1** | 19E | 223015 | 2.90 | N.A. | **122±7** | 14±5 | 7±11 | 9.2±0.7 | N.A. | 9.5±0.9 | 712±18 | 2.30±0.12 | 3.24±0.13 | N.A. | N.A. | N.A. | 395±15 | 0 | 10 |
| **19E-2** | 19E | 223016 | 2.40 | N.A. | **97±5** | 15±5 | 16±3 | 17.0±0.4 | 20±4 | 18.8±0.4 | 773±10 | 2.70±0.13 | 3.64±0.15 | N.A. | N.A. | N.A. | 353±9 | 1 | 9 |
| **19E-3** | 19E | 223017 | 2.15 | N.A. | **117±7** | 14±5 | -4±10 | 10.5±0.7 | N.A. | 12.2±0.8 | 750±17 | 2.48±0.12 | 3.41±0.14 | N.A. | N.A. | N.A. | 400±14 | 1 | 9 |
| **19E-4** | 19E | 223018 | 0.90 | N.A. | **92±5** | 15±5 | 1±18 | 15.1±1.2 | N.A. | 12.6±1.4 | 672±23 | 2.35±0.12 | 3.29±0.14 | N.A. | N.A. | N.A. | 302±7 | 1 | 9 |
| **19L-1** | 19L | 223001 | 1.45 | N.A. | **>260** § | 13±5 | 18±5 | 10.3±0.4 | N.A | 17.3±0.5 | 945±16 | 3.09±0.15 | 4.03±0.17 | N.A. | N.A. | N.A. | 1066±61** | 0 | 3 |
| **19L-2** | 19L | 223002 | 3.50 | N.A. | **>250** § | 15±5 | 21±8 | 13.0±0.6 | N.A | 16.2±0.6 | 963±16 | 3.07±0.15 | 4.01±0.16 | N.A. | N.A. | N.A. | 997±36** | 0 | 3 |
| **19L-3** | 19L | 223003 | 4.45 | N.A. | **>210** § | 14±5 | 17±4 | 17.0±0.7 | 15±5 | 21.6±0.6 | 999±15 | 3.29±0.17 | 4.23±0.18 | N.A. | N.A. | N.A. | 887±18** | 0 | 3 |
| **19L-4** | 19L | 223004 | 5.75 | N.A. | **>270** § | 15±5 | 14±2 | 14.1±0.2 | 17±2 | 15.5±0.2 | 945±7 | 2.97±0.15 | 3.91±0.16 | N.A. | N.A. | N.A. | 1076±49** | 0 | 3 |
| **19M-1** | 19M | 223009 | 2.25 | N.A. | **>220** § | 16±5 | 42±4 | 36.6±0.7 | 41±5 | 55.3±0.6 | 775±11 | 3.47±0.17 | 4.11±0.18 | N.A. | N.A. | N.A. | 899±25** | 0 | 3 |
| **19M-2** | 19M | 223010 | 1.37 | N.A. | **>240** § | 11±5 | 23±7 | 11.7±0.6 | N.A | 21.6±0.6 | 697±13 | 2.57±0.13 | 3.51±0.14 | N.A. | N.A. | N.A. | 827±73** | 0 | 3 |
| **19K-1** | 19K | 223019 | 2.80 | N.A. | **>250** § | 15±5 | 21±4 | 18.2±0.5 | 28±5 | 23.3±0.5 | 599±11 | 2.31±0.11 | 3.25±0.13 | N.A. | N.A. | N.A. | 819±50** | 0 | 3 |
| **C-1** | C | 223020 | 6.25 | N.A. | **94±5** | 15±5 | 8±9 | 17.6±1.5 | 23±10 | 10.3±1.2 | 472±21 | 1.74±0.10 | 2.67±0.11 | N.A. | N.A. | N.A. | 252±8 | 1 | 10 |
| **C-2** | C | 223021 | 4.9 | N.A. | **103±6** | 10±5 | 15±5 | 11.7±1.1 | 12±6 | 8.9±0.8 | 395±14 | 1.53±0.08 | 2.47±0.10 | N.A. | N.A. | N.A. | 254±9 | 1 | 10 |
| **D-1** | D | 223022 | 5.15 | N.A. | **104±6** | 10±5 | 12±6 | 9.9±1.0 | 8±7 | 12.9±0.8 | 833±19 | 2.75±0.15 | 3.68±0.16 | N.A. | N.A. | N.A. | 383±9 | 0 | 11 |
| **D-2** | D | 223023 | 4.7 | N.A. | **89±5** | 10±5 | 35±7 | 23.1±1.5 | 22±8 | 36.2±1.2 | 702±21 | 2.96±0.16 | 3.90±0.17 | N.A. | N.A. | N.A. | 346±9 | 1 | 13 |

| | | | | | | | | | | | | | | | | | | | |
|---|---|---|---|---|---|---|---|---|---|---|---|---|---|---|---|---|---|---|---|
| **D-3** | D | 223024 | 3.75 | N.A. | **116±7** | 10±5 | 12±6 | 16.6±1.0 | 21±7 | 20.8±0.9 | 785±19 | 2.86±0.15 | 3.80±0.16 | N.A. | N.A. | N.A. | 442±15 | 1 | 10 |
| **D-4** | D | 223025 | 2.8 | N.A. | **101±6** | 10±5 | 19±6 | 14.2±1.2 | 6±7 | 17.4±0.9 | 854±21 | 2.98±0.16 | 3.91±0.17 | N.A. | N.A. | N.A. | 395±13 | 0 | 11 |
| **17I-1** | 17I | 193014 | 3.3 | >84 * | **128±9** | 12±5 | 22±9 | 21.3±1.5 | 23±10 | 12.7±1.2 | 612±23 | 2.35±0.13 | 3.22±0.14 | 196±9 | 0 | 12 | 418±19 | 0 | 7 |
| **17I-2** | 17I | 193015 | 2 | >85 * | **117±9** | 15±5 | 34±10 | 23.4±1.6 | 25±11 | 18.9±1.3 | 862±29 | 3.05±0.16 | 3.93±0.17 | 258±9 | 0 | 15 | 463±27 | 0 | 6 |

Notes:

(1) Ages denoted with an asterisk (*) are minimum ages because the $D_e$ values are >150 Gy and K-feldspar $pIRIR_{200,290}$ ages (bold) are used for interpretation in the main text.

(2) All grain sizes were 180-250 µm except for K-rich feldspar from samples 193002 and 203009 for which it was 90-180 µm.

(3) A residual dose of 19±5 Gy was subtracted from K-feldspar $pIRIR_{200,290}$ $D_e$ values prior to age calculation. The $pIRIR_{200,290}$ dose values denoted with double asterisk (**) are averages of 2x$D_c$ values; these are used to calculate minimum ages (denoted with §) because the natural pIRIR signals were in saturation. $D_c$ is the characteristic dose value obtained when fitting the dose response curve with a single saturating exponential function ($I = I_{sat}$ x (1-exp(D/$D_c$)) in which I is sensitivity-corrected light level, $I_{sat}$ is sensitivity-corrected light level at saturation and D is dose. When D=2x$D_c$, I=$I_{sat}$x0.86.

(4) Note that samples C1, C2, D1-D4, 17I-1 and 17I-2 (shaded gray) were also dated at the Xi'an lab using quartz OSL (Supplementary Table 3).

(5) The interquartile range (IQR) criterion was used to reject outliers from the $D_e$ distributions. $D_e$ values that were lower than Q1-1.5xIQR or higher than Q3+1.5xIQR were rejected and the number of rejected aliquots denoted with '$n_r$'. The total number of accepted aliquots is given under '$n_a$'.

**Supplementary Table 5. Data on river meander wavelength and discharge in China**

| River | Gauge name | Longitude | Latitude | Meander wavelength (m) | Mean annual discharge ($10^8$ $m^3$) | Years of discharge | Discharge data source |
|---|---|---|---|---|---|---|---|
| Kherlen R. | Alatan | 116.820 | 48.648 | 400 | 4.83 | 1963-2007 | Ref. 18 |
| Wuerxun R. | Kunduleng | 117.750 | 48.067 | 497 | 6.26 | 1963-2007 | Ref. 18 |
| Hailar R. | Bahou | 119.731 | 49.263 | 1514 | 33.58 | 1956-2006 | Ref. 19 |
| Yimin R. | Hailar | 119.745 | 49.187 | 1008 | 11.62 | 1956-2006 | Ref. 19 |
| Yimin R. | Honghuaerji | 119.983 | 48.283 | 621 | 7.54 | 1960-2006 | Ref. 19 |
| Hailar R. | Yakeshi | 120.677 | 49.333 | 969 | 20.8 | 1956-2006 | Ref. 19 |
| Kuduer R. | Wuerqihan | 121.367 | 49.567 | 454 | 4.93 | 1960-2006 | Ref. 19 |
| Yimin R. | Yiminmuchang | 119.800 | 48.650 | 738 | 9.34 | 1959-2006 | Ref. 19 |
| Hui R. | Huihekou | 119.723 | 49.027 | 160 | 1.25 | 1956-2006 | Ref. 19 |
| Moleger R. | Touzhan | 119.582 | 49.500 | 217 | 1.4 | 1956-2006 | Ref. 19 |
| Hailar R. | Wangong | 118.909 | 49.209 | 1892 | 34.74 | 1956-2006 | Ref. 19 |
| Songhua R. | Jiamusi | 130.365 | 46.820 | 10663 | 631.29 | 1956-2014 | Ref. 20 |
| Neng R. | Dalai | 124.263 | 45.547 | 4845 | 208.4 | 1956-2014 | Ref. 20 |
| Songhua R. | Songhuajiang | 125.915 | 44.766 | 4224 | 144 | 1956-2010 | Ref. 21 |
| Neng R. | Jiangqiao | 123.695 | 46.792 | 4213 | 202.44 | 1955-2010 | Ref. 22 |
| Neng R. | Kumotun | 125.265 | 49.446 | 2372 | 55.13 | 1955-2005 | Ref. 22 |
| Miandu R. | Daqiaotun | 120.756 | 49.279 | 644 | 8.9509 | 1960-2006 | Ref. 19 |
| Yellow R. | Maqu | 102.084 | 33.960 | 2877 | 140.36 | 1952-2008 | Ref. 23 |
| Huangshui R. | Liancheng | 102.817 | 36.600 | 1580 | 27.03 | 1956-2008 | Ref. 23 |
| Huai R. | Xixian | 114.734 | 32.326 | 1932 | 37.96 | 1951-2010 | Ref. 24 |
| Huai R. | Wangjiaba | 115.599 | 32.429 | 3325 | 95.1 | 1951-2010 | Ref. 24 |
| Wei R. | Huaxian | 109.761 | 34.583 | 3000 | 64.65 | 1960-2009 | Ref. 25 |
| Yellow R. | Toudaoguai | 111.063 | 40.268 | 6920 | 286 | 1964-2015 | Ref. 26 |
| Yellow R. | Gaocun | 115.080 | 35.388 | 10156 | 330 | 1955-2016 | Ref. 27 |

## Supplementary References


1. Wünnemann, B. & Hartmann, K. Morphodynamics and paleohydrography of the Gaxun Nur Basin, Inner Mongolia, China. *Zeitschrift fur Geomorphologie N.H.* **126**, 147-168 (2002).

2. Li, G. et al. Quartz and K-feldspar luminescence dating of a Marine Isotope Stage 5 megalake in the Juyanze Basin, central Gobi Desert, China. *Palaeogeogr. Palaeoclimatol. Palaeoecol*. **440**, 96-109 (2015).

3. Fan, Y. et al. Potential water sources for Late Quaternary Megalake Jilantai-Hetao, China, inferred from mollusk shell 87Sr/86Sr ratios. *J. Paleolimnol.* **43**, 577-587 (2010).

4. Chen, F. et al. Landscape evolution of the Ulan Buh Desert in northern China during the late Quaternary. *Quat. Res.* **81**, 476-487 (2014).

5. Zhang, H. et al. Chronology of the shell bar section and a discussion on the ages of the Late Pleistocene lacustrine deposits in the paleolake Qarhan, Qaidam basin. *Front. Earth Sci. China* **2**, 225-235 (2008).

6. Lai, Z., Mischke, S. & Madsen, D. Paleoenvironmental implications of new OSL dates on the formation of the "Shell Bar" in the Qaidam Basin, northeastern Qinghai-Tibetan Plateau. *J. Paleolimnol.* **51**, 197-210 (2014).

7. Gu, X. et al. Macroinvertebrates community structure and water quality assessment in the North Canal River Basin, Beijing, China. *J. Lake Sci.* **29**, 1444-1454 (2017).

8. Li, J. et al. Diversity of benthic macroinvertebrates in Luanhe River Basin in summer. *Chin. Agric. Sci. Bull.***31**, 40-50 (2015).

9. Wang, Q. & Chang, Y. Reproductive biology of Asian Clam *Corbicula fluminea* in Dayang River in Liaoning Province. *J. Dalian Fisheries Univ.* **25**, 8-13 (2010).

10. Xie, S., Huang, B., Wang, H., Song, C. & Shi, B. Assessment of water quality in Baiyangdian Lake by zoobenthos Biodiversity. *J. Hydroecol*. **3**, 43-48 (2010).

11. Zhao, X. et al. The reproductive biology of *Corbicula fluminea* from the Yellow River Delta. *Oceanol. Limnol. Sin.* **43**, 1008-1015 (2012).

12. Zhou, H. et al. Shell morphology of *Corbicula fluminea* (Müller, 1774) and its implication for the adaptation to environmental change in the major drainage basins of China. *Chinese J. Ecol.* **30**, 1497-1503 (2011).

13. Li, G. et al. Quantitative precipitation reconstructions from Chagan Nur revealed lag response of East Asian summer monsoon precipitation to summer insolation during the Holocene in arid northern China. *Quat. Sci. Rev.* **239**, 106365 (2020).

14. Goldsmith, Y. et al. Northward extent of East Asian monsoon covaries with intensity on orbital and millennial timescales. *Proc. Natl Acad. Sci. USA* **114**, 1817-1821 (2017).

15. Yang, D. et al. Analyzing spatial and temporal variability of annual water-energy balance in nonhumid regions of China using the Budyko hypothesis, *Water Resour. Res.* **43**, W04426 (2007).

16. Huo, J. et al. Effects of watershed char and climate variables on annual runoff in different climatic zones in China, *Sci. Total Environ.***754**, 142157 (2021).

17. Abatzoglou, J. et al. TerraClimate, a high-resolution global dataset of monthly climate and climatic water balance from 1958–2015. *Sci. Data* **5**, 170191 (2018).

18. Huang, J. *Stochastic Analysis of Hydrological Time Series Based on Wavelet Theory in Hulun Basin* (Master thesis, Inner Mongolia Agricultural University, Huhhot, 2011).

19. Duan, L., Liu, T., Wang, X., Luo, Y. & Wu, L. Development of a regional regression model for estimating annual runoff in the Hailar river basin of China. *J.Water Resour. Prot*. **2**, 934 (2010).

20. Wang, X. et al. Variation characteristics of annual runoff in the Songhua River Basin from 1956 to 2014. *Soil Water Conserv. China* 61-65 (2017).

21. Zhang, C. & Yu, D. Songhua River hydrological station runoff trend analysis. *Jilin Water Resour.* 36-40 (2015).

22. Wang, Y., Wang, S. & Su, T. Contributions of precipitation and hunman activities to runoff change in the Songhua River Basin. *J. Nat. Resour.* **30**, 304-314 (2015).

23. Wang, J. & Hu, X. Runoff Space-time distribution rule and evolution tendency of Yellow River unstream and main tributaries. *J. China Hydrol*. **31**, 90-96 (2011).

24. Wang, G., Wang, Y. & Zhang, M. Runoff variations and its response to the precipitation changes in Yellow-Huai River Basin. *Yellow River* **36**, 52-54 (2014).

25. Hou, Q., Bai, H., Ren, Y., He, Y. & Ma, X. Analysis of variation in runoff of the main stream of the Weihe River and related driving forces over the last 50 years. *Resour. Sci.* **33**, 1505-1512 (2011).

26. Qiang, G. *Analysis of Runoff Variation Characteristics and Forecast in Ningxia-Inner Mongolia Reach of the Upper Yellow River* (Master thesis, Lanzhou University of Technology, Lanzhou, 2017).

27. Wang, X., Engel, B., Yuan, X. & Yuan, P. Variation analysis of streamflows from 1956 to 2016 along the Yellow River, China. *Water* **10**, 1231 (2018).
